# Non-Islet Cell Tumor Hypoglycemia: A Rare Cause of Hypoglycemia in Pulmonary Sarcomatoid Cancer

**DOI:** 10.7759/cureus.1972

**Published:** 2017-12-20

**Authors:** Timothy B Legare, Oteni Hamilton, Sarah Dhannoon, Sayed Ali

**Affiliations:** 1 College of Medicine, University of Central Florida College of Medicine; 2 Internal Medicine Residency, University of Central Florida College of Medicine; 3 Medicine, Orlando VAMC

**Keywords:** nicth, hypoglycemia, lung cancer

## Abstract

Non-islet cell tumor hypoglycemia (NICTH) is a rare paraneoplastic condition caused most commonly by metastatic mesenchymal tumors. A 74-year-old non-diabetic male with an eight-year history of metastatic sarcomatoid lung cancer presented with altered mental status. His previous treatment included a lobectomy and radiation. Laboratory investigations were significant for blood glucose of 28 mg/dL, confirming hypoglycemia. He was hypokalemic, a condition seen in approximately 50% of patients with NICTH, at 2.8 mEq/L of potassium (normal 3.5-5.2 mEq/dL) and his urine toxicology screen was negative. A computed tomography (CT) of the head was negative for any acute events. His tumor burden had progressed as compared to previous CTs. Administration of dextrose resolved his symptoms. Laboratory studies during subsequent hypoglycemic events measured his insulin-like growth factor-I (IGF-I) at 51 ng/mL, insulin-like growth factor-II (IGF-II) at 290 ng/mL, growth hormone (GH) at 0.6 ng/mL, C-peptide at 0.16 ng/mL (low), and insulin levels at <1 uIU/mL. ‘Big’ IGF-II, the gold standard for the diagnosis of NICTH, was not available at our facility. Based on these results, NITCH was diagnosed clinically. NICTH is a rare condition with episodes of recurrent hypoglycemic episodes in the setting of metastatic cancer. Ideal therapy for NICTH is tumor resection or debulking. In cases of inoperable tumors, glucocorticoids or recombinant human growth hormone (rGH) maintain euglycemia, with glucagon rescue in case of an emergency.

## Introduction

Hypoglycemia is a life-threatening condition with a multitude of causes. Cancer-causing hypoglycemia is broadly categorized into three distinct areas. Hypoglycemia can manifest when a tumor produces insulin as in pancreatic or ectopic insulinomas, when a tumor infiltrates the liver or adrenal glands and causes local destruction, or when tumors produce a paraneoplastic disorder by the production of substances that activate receptors causing hypoglycemia. Non-islet cell tumor hypoglycemia (NITCH) is one such rare paraneoplastic disorder that is characterized by tumor production of partially processed insulin-like growth factor-II (termed ‘big’-IGF-II) [1]. The tumors associated with NICTH are usually of mesenchymal origin [2]. Here, we present a case of NICTH in a patient with metastatic sarcomatoid carcinoma of the lung.

## Case presentation

**Figure 1:**
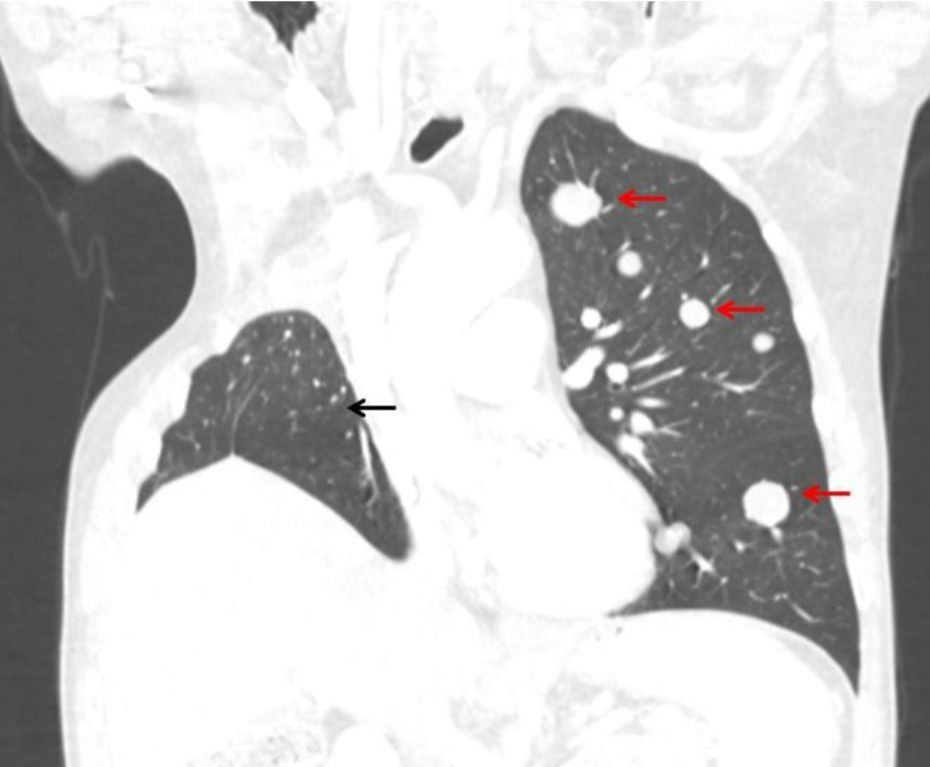
Computed tomography of the chest This is a coronal view of the patients CT chest in the preset lung window. The red arrows demonstrate metastatic lesions of pulmonary sarcomatoid lung cancer in the left lung. The black arrow demonstrates the patients' remaining right lower lobe of the lung status, post a right upper lobe resection.

A 74-year-old non-diabetic male presented to the emergency department with acute onset altered mental status. His past medical history was significant for metastatic sarcomatoid lung cancer following a right upper lobe resection of a 15 cm x 13 cm primary tumor with positive margins. Later, he was found to have metastatic lesions in the left lung, pararenal retroperitoneal compartments, right trapezius, left obturator internus, right pectineus, right hamstring muscles, and the gastrohepatic ligament, which were treated with palliative radiation. The most recent chest computed tomography (CT) is presented below in Figure 1, demonstrating a right upper lobe resection and metastatic lesions in the left lung.

He had previously deferred chemotherapy. He had been resting at home when he abruptly stood from his chair and began to speak nonsensical words to his wife. His speech was similar to a receptive aphasia, with sentences described as “Potato. Word. Image. Done.” He was unresponsive to questions but would mimic the movements of his wife. Additionally, he had profound global weakness during this event, needing both his wife and a neighbor to transport him to the hospital. Upon arrival at the emergency department, a head CT ruled out an acute bleed, his urine drug screen was negative, and his wife denied the use of any over-the-counter medications and herbal supplements. His finger-stick glucose was <45 mg/dL. He was given one liter of 5% dextrose in normal saline with immediate correction of his altered mental status. Initial laboratory investigation, prior to his dextrose correction in the emergency room upon presentation, showed hypokalemia at 2.5 mEq/dL (normal 3.5-5.2 mEq/dL), serum glucose at 28 mg/dL, and hemoglobin A1c (HbA1c) at 5.3%. During his admission, he continued to have hypoglycemic episodes, despite being monitored for caloric intake and medication administration. During one of his hypoglycemic events, he was symptomatic with a blood sugar of 64 mg/dL, blood was redrawn for insulin at <1.0 ulU/mL, C-peptide at 0.16 ng/mL (normal: 0.80-3.85 ng/mL), growth hormone (GH) at 0.6 ng/mL (normal: < 7.1 ng/mL), insulin-like growth factor-I (IGF-I) at 37 ng/mL (Z-Score: -1.3, normal: 34-246 ng/mL), and insulin-like growth factor-II (IGF-II) at 290 ng/mL (normal: 267-616 ng/mL). His ratio of IGF-II to IGF-I was approximately 8:1 and the ratios of IGF-II to IGF-I greater than 10:1 are highly suggestive for NICTH in the setting of an inability to measure IGF-II [3]. He was diagnosed clinically with NICTH based upon his recurrent hypoinsulinemic hypoglycemic episodes and started on oral prednisone 30 mg twice daily. He was discharged with instructions for monitoring hypoglycemia, education on glucagon rescue injection, and close follow-up with both his primary care provider and oncologist. The patient improved clinically on oral steroids. At the three-month follow-up, he required increasing doses of steroids to control his hypoglycemia, as his tumor burden increased. He continued to defer chemotherapy and any surgical treatment option.

## Discussion

The pathophysiology of NICTH is due to the production of under-processed IGF-II, named the ‘big’-IGF-II protein. The proposed mechanism of increased ‘big’-IGF-II leading to hypoglycemia is that the elevated levels of ‘big’-IGF-II displace IGF-I and normally processed IGF-II from the insulin-like growth factor binding proteins (IGFBPs) causing increased free insulin-like growth factors (IGFs) [1]. The free IGF proteins act in an insulin-like manner, causing increased peripheral uptake of glucose in both muscle and adipose, decreased lipolysis, and decreased free glucose production by the liver. In addition to the effects of increased IGFs causing the peripheral utilization of glucose, there is also negative biofeedback via the elevated IGF to suppress the production of growth hormone (GH), further exacerbating the hypoglycemia [1]. The specific diagnosis of NICTH is based upon the values of the serum concentration of ‘big’-IGF-II via an immunoblot assay and mature IGF-II via tricine sodium dodecyl sulfate-polyacrylamide gel electrophoresis (SDS-PAGE) analysis [1]. These are proposed as the most reproducible and sensitive tests for distinguishing ‘big’-IGF-II from mature IGF-II [4]. Our hospital system did not have access to these tests and relied on a clinical diagnosis of NICTH. A serum ratio of IGF-II:IGF-I that is greater than 10 to one is indicative of NICTH but is not seen in all patients [3]. Additionally, hypokalemia was found in 25 of 47 (53%) patients diagnosed with NICTH [5]. Even though the confirmatory column chromatography of ‘big’-IGF-II was not ordered, which is the “gold standard,” a clinical diagnosis of NICTH can still be made. In a review of the literature in 2013, the authors suggest that a clinical diagnosis be made in patients with an oncologic disease who present with hypoinsulinemic hypoglycemia and laboratory evidence of a low IGF-I, a normal total IGF-II, and a low GH [3].

The treatment of NICTH is currently best achieved with the resection or debulking of the tumor burden [1]. In cases of inoperable tumors, as seen in our patient, low, moderate, or even high doses of oral glucocorticoids have been effective in stabilizing blood sugar and reducing hypoglycemic events [6-7]. The physiological mechanism of how glucocorticoids influence the pathophysiology of NICTH is not completely elucidated. It has been shown that glucocorticoid therapy reduces the serum levels of ‘big’-IGF-II, but the mechanism is not clear. It is proposed that the glucocorticoid therapy either suppresses tumor production of ‘big’-IGF-II or increases clearance from the serum [8]. Another therapy that has shown effectiveness in the treatment of NICTH has been the use of human recombinant growth hormone (hGH) [6,8-9]. The mechanism of hGH in suppressing hypoglycemic episodes is increasing IGF binding protein 3 (IGF BP3) and acid labile subunit (ALS), which decrease circulating ‘big’-IGF-II while also stimulating hepatic gluconeogenesis [8,10]. The effectiveness of hGH has been shown to reduce the hypoglycemic episodes but did not raise insulin levels as was seen with glucocorticoids [8]. Ultimately, the optimal therapy for patients with disease that cannot be treated surgically or debulking may be a combination treatment of glucocorticoids and hGH.

## Conclusions

This case report concludes that a patient with repetitive hypoinsulinemic hypoglycemic episodes should be evaluated for NICTH. Additionally, the treatment of NICTH can be accomplished with oral glucocorticoids for the maintenance of euglycemia if the risks and benefits have been discussed with the patient.
